# From imagination to activism: Cognitive alternatives motivate commitment to activism through identification with social movements and collective efficacy

**DOI:** 10.1111/bjso.12811

**Published:** 2024-11-15

**Authors:** Julian Bleh, Torsten Masson, Sabrina Köhler, Immo Fritsche

**Affiliations:** ^1^ Leipzig University Leipzig Germany

**Keywords:** collective action, politicized identity, self‐efficacy, social change, social identity, utopian thinking, visions

## Abstract

Having a vision and being able to imagine socially and ecologically just alternatives can motivate people for societal transformation. However, which psychological processes drive this link between the mental accessibility of societal alternatives and collective action? We hypothesized that collective efficacy beliefs and politicized identification form two pathways mediating the effects of cognitive alternatives on high‐cost activist behaviour. Two studies and a pooled analysis tested these hypotheses longitudinally. Data were collected in two field settings: a climate camp and an online conference on socio‐ecological visions. In line with our assumptions, and across three of the four analysed timeframes, latent change score modelling showed that changes in cognitive alternatives predicted changes in collective efficacy beliefs and social movement identification, which in turn, predicted changes in collective action intentions. We found clear evidence for our hypotheses in the short term and mixed evidence in the long term. Additional analyses including participative efficacy showed no relevant effects. We concluded that the ability to envision social change may foster a sense of agency as members of social movements. These processes linking imagination to activism are less about individual efficacy than about realizing the collective possibilities for change and identifying with the groups enacting it.

## INTRODUCTION

*‘*We are unstoppable, another world is possible*’* has been a rallying cry of various social movements since the 1990s (e.g., Gralke, 2022; Manski et al., 2020; Ng & Khan, 2012). The slogan symbolizes how the ability to envision alternatives to the status quo is linked to the perception of collective agency. It reflects an influential idea in social theory: our capacity for societal imagination lies at the heart of social change (e.g., Bloch, 1985; Mannheim, 2003; Wright, 2010; Zittoun & Gillespie, 2018). This capacity might have never been more important than today (Costanza, 2000; Moore & Milkoreit, 2020): *having a vision* is important for pursuing any kind of change but indispensable for transformational change, such as the shift to a socially and ecologically just society (Milkoreit, 2017). A growing body of research indicates that the imagination of desirable societies can increase support for social change (Bain et al., 2013; Bosone et al., 2023; Daysh et al., 2024; Fernando et al., 2018, 2020; Wright et al., 2020, 2022). However, little is known about the psychological processes that underlie this effect. In this article, we investigate these processes longitudinally by testing two pathways from imagination to activism. We propose that being able to imagine a better society motivates activism via collective efficacy beliefs and politicized identification. Before exploring the theoretical underpinnings of these two pathways, we first review research on the motivational effects of the mental accessibility of societal alternatives.

### Cognitive alternatives: The mental accessibility of social change

Imagining a desirable society seems to motivate action for the collective (Bosone et al., 2023; Fernando et al., 2018, 2020). In a series of experiments, Fernando et al. (2018) asked people to imagine and describe their ideal society. Compared to reflecting on the present society, this *utopian thinking* task increased people's criticism of the status quo and their readiness to act for social change. Recently, studies have shown similar results, specifically for pro‐environmental action: Imagining a utopian climate‐friendly society increased people's intentions to engage in collective climate action, both directly and indirectly via increased feelings of hope (Daysh et al., 2024). Moreover, reading a positive vision about a decarbonated ecological society motivated participants to engage in pro‐environmental action (Bosone et al., 2023). Also, it increased (environmental) *cognitive alternatives*, the mental accessibility of desirable alternatives to the status quo (see Tajfel & Turner, 1979; Wright et al., 2020). This self‐reported ability to envision what a sustainable world could be like was associated with a higher willingness to engage in environmental activism and an increased likelihood to write a letter to the Ministry of the Environment (Wright et al., 2022).

**FIGURE 1:**
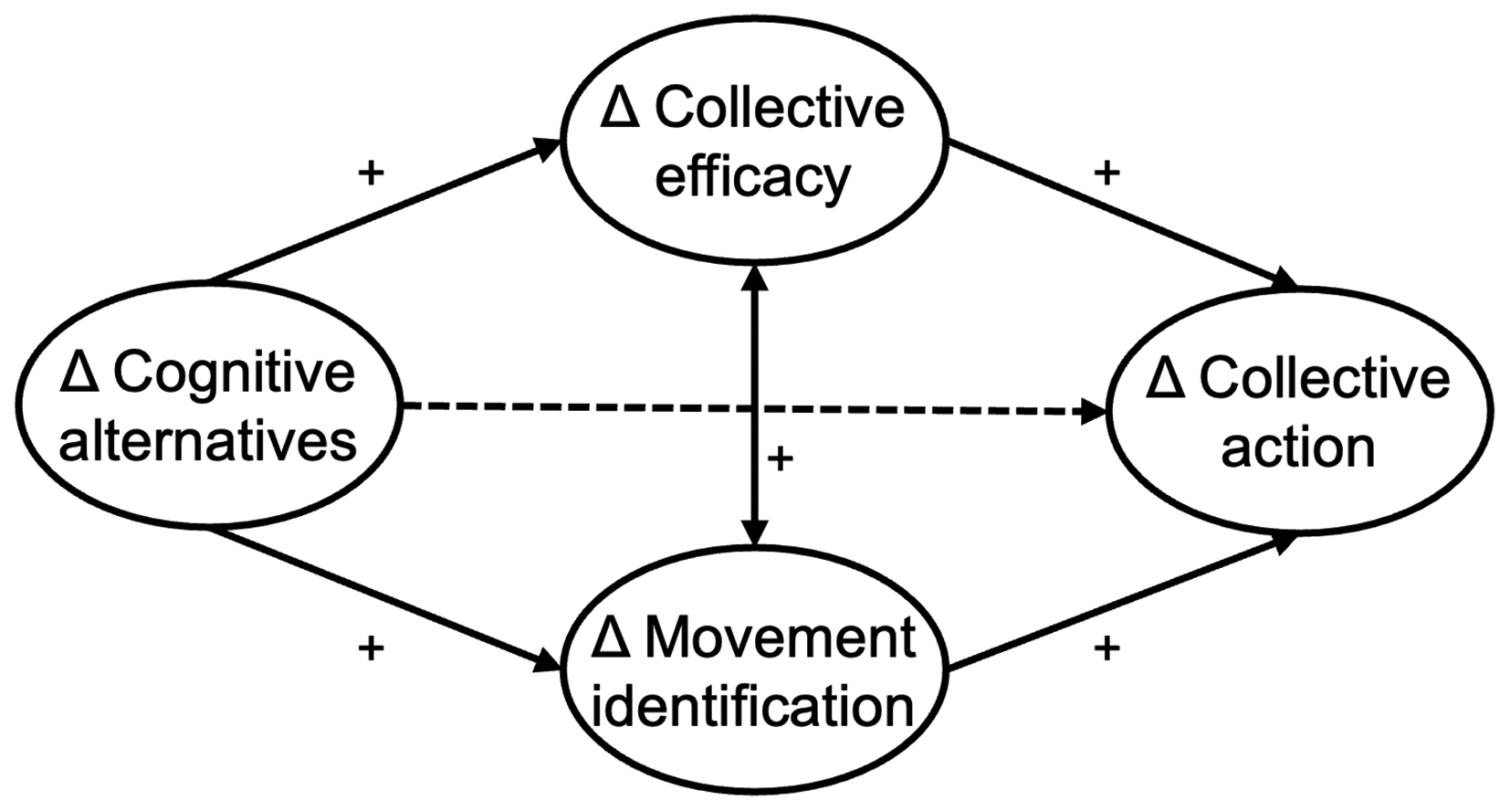
Conceptual model: Changes in cognitive alternatives positively predict changes in collective action, mediated by changes in collective efficacy and movement identification. There is no direct link between changes in cognitive alternatives and collective action.

How can this link between the ability to imagine societal alternatives and the motivation to engage in social change be explained? Building on models of collective (environmental) action (Agostini & Van Zomeren, 2021; Fritsche et al., 2018; Fritsche & Masson, 2021; Thomas et al., 2011; Van Zomeren et al., 2008), identification with an agentic ingroup is crucial for individuals' motivation to engage in social change. Involvement in a collective project may turn personal helplessness and inertia in the face of large‐scale social and environmental crises into a sense of group‐based control and motivate people to engage in collective action (Fritsche, 2024). We suggest that the mental accessibility of desirable societal alternatives can lead to social change activism by strengthening social movement members' perception of being part of (i.e., identifying with) a group that has the capability to impose social change (i.e., collective efficacy; see Figure 1 for conceptual model).

### Two pathways from imagination to activism

Models of collective action, such as the social identity model of collective action (see also Thomas et al., 2011; SIMCA; Van Zomeren et al., 2008) and the social identity model of pro‐environmental action (SIMPEA; Fritsche et al., 2018), agree upon collective efficacy and collective identification as being main drivers of collective action (alongside injustice beliefs [Van Zomeren et al., 2008] and social norms [Fritsche et al., 2018]). In the following, we will focus on these two central components that give rise to an agentic social self (Fritsche, 2024).

#### Efficacy path

We suggest that one path from imagination to activism rests on the perception of efficacy, especially *collective efficacy*, a person's belief that their group can affect its relevant environment (e.g., society) in a desired way (Bandura, 1997; Hamann et al., 2024). Being able to imagine desirable social change should correspond with an increased perception that change is attainable by collective effort. This is because salient alternatives to the status quo might work as an anchor or simulation heuristic (Kahneman & Tversky, 1982; Tversky & Kahneman, 1974) of how possible these alternatives are and how likely the group is collectively efficacious in bringing about these changes (Cervone & Peake, 1986). At the same time, due to collective self‐serving bias, people might be inclined to attribute probable positive societal changes to activities of their own collective (Sherman & Kim, 2005; Taylor & Doria, 1981). Thus, picturing how society *could be* can lead people to realize that change through their social self *is possible*. In fact, initial evidence from laboratory studies demonstrated that reading a positive vision (vs. no task) increased collective efficacy beliefs and collective action intentions (Bosone et al., 2023) and that efficacy beliefs mediated the effect of imagining a green sufficiency society (vs. a high‐tech abundance society) on social change motivation (Fernando et al., 2020). Moreover, a field study among student‐led sustainability initiatives showed cross‐sectionally that having a vision of what a sustainable university could look like was positively associated with efficacy beliefs regarding the successful implementation of such changes (Hamann et al., 2021). We thus expect that an increase in the ability to imagine social change strengthens collective efficacy beliefs, which in turn motivate the collective pursuit of a better society.

#### Identification path

The second path from imagination to activism is based on politicized identification, that is, self‐categorization as a member of a group that actively promotes (at least supports) social change (Simon & Klandermans, 2001). Identifying with activist groups or social movements is a very powerful and proximal predictor of collective action (Schmitt et al., 2019; Simon et al., 1998; Van Zomeren et al., 2008). We propose that the ability to imagine social change strengthens one's identification with corresponding movements. Social movements are generally trying to promote (or prevent) social change based on a shared normative understanding of how society *should* *be* (Della Porta & Diani, 1999; Wathne, 2023). Consequently, being able to imagine desirable alternatives to the status quo should raise people's awareness of their personal membership in movements whose norms align with their imaginations of a better society. Preliminary evidence supports this reasoning. Using cross‐sectional data, Wright et al. (2022) found that the relationship between environmental cognitive alternatives and activist behaviour was mediated by identification with environmental activists. We thus expect that a heightened ability to imagine a better society boosts one's identification with social change movements, which, in turn, increases the intention to participate in change‐directed collective action.

### The present research

The present research is the first longitudinal investigation of the relationship between having a vision and collective action for social change. Our hypotheses are that collective efficacy beliefs and social movement identification form two unique pathways linking the ability to imagine social change to change‐directed collective action over time (Figure 1). Similar to Wright et al. (2020, 2022), we have operationalized the ability to imagine social change as the accessibility of cognitive alternatives. Extending Wright and colleagues' focus on environmental cognitive alternatives, our approach includes both social as well as ecological alternatives to the status quo. This notion of *socio‐ecological cognitive alternatives* is in line with Wright et al. (2020) reasoning that the accessibility of environmental cognitive alternatives also involves one's ability to imagine different status relationships in other (non‐environmental) areas of society.

To the best of our knowledge, the current research is also the first to investigate the ability to imagine social change among members of social movements, assessing changes in cognitive alternatives as an outcome of social movement activities. It examines how these changes are translated into high‐cost activist behaviour, such as poorly or completely unpaid work, regular participation in protests, and civil disobedience, irrespective of the unpleasant personal consequences these activities might have. The value of this approach lies in testing the generalizability of previous findings from laboratory studies in relevant field settings and samples. We surveyed the participants of a climate camp (Study 1) and an online conference on socio‐ecological visions (Study 2). Both settings provided participants with the opportunity to actively imagine, practice, and disseminate alternatives to the status quo (see *prefigurative politics*; e.g., Cornish et al., 2016). This allowed us to measure changes in cognitive alternatives and examine how these changes relate to changes in collective efficacy beliefs, politicized identification, and subsequent changes in collective action intentions.

Both studies were part of larger longitudinal data collections (for more details, see https://osf.io/ytz6c/ [Study 1] and https://osf.io/8chn3/ [Study 2]). Data were collected at three time points: directly before (T1) and after participation (T2) in the week‐long events, as well as in a follow‐up measurement (T3) five (Study 1) or 13 months (Study 2) later. We expect changes in cognitive alternatives (T2–T1, T3–T1) to positively predict (a) changes in collective efficacy beliefs (T2–T1, T3–T1) and (b) changes in social movement identification (T2–T1, T3–T1). In turn, changes in collective efficacy beliefs and movement identification are expected to positively predict changes in collective action intentions (T2–T1, T3–T1). Overall, there should be two positive indirect effects of cognitive alternatives on collective action through collective efficacy and movement identification.

#### Analysis strategy

**FIGURE A1:**
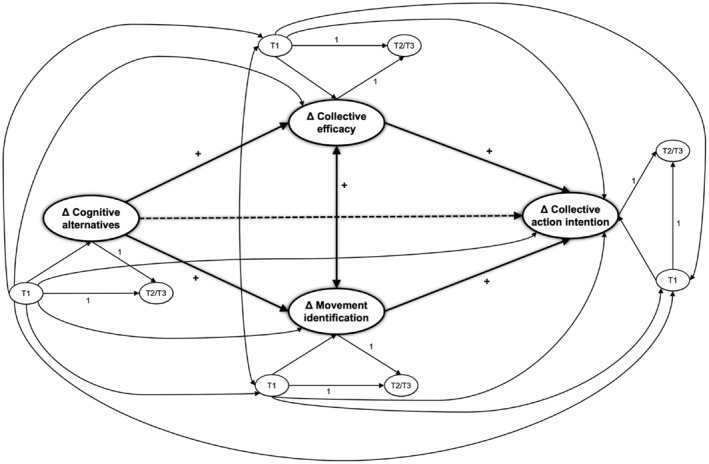
Parallel latent change score mediation model including all regression and covariation paths. For ease of display, indicators, residuals, variances, and intercepts are not included in the figure. Thick lines have hypotheses: ‘+’ indicates that a positive relationship is expected. Dashed lines indicate that no significant positive relationship is expected. Thin lines do not have hypotheses.

We used latent change score (LCS) modelling to capture such within‐person changes (also denoted by Δ) as error‐free change estimates between two time points (Castro‐Schilo & Grimm, 2018): pre–post (T2–T1) and pre‐follow‐up (T3–T1) changes. This allows us to examine how changes in one variable predict changes in another variable while controlling for baseline levels (Selig & Preacher, 2009).^1^ For testing the conceptual model (Figure 1, also see Figure A1 in Appendix), we first investigated the bivariate correlations between the change scores of all study variables. Next, we tested the hypothesized mediation paths as separate simple mediation models and, finally, as parallel models including both mediators. In Study 2, we added participative efficacy beliefs to explore what kind of efficacy beliefs mediate the relationship between cognitive alternatives and collective action. Due to sample size constraints and to address cross‐loadings, we reduced model complexity by applying item parcelling to some of our measures (Rioux et al., 2020).

Analyses were conducted using the lavaan R‐package (version 0.6–17). We based our syntax for the construction of latent change score models on Kievit et al. (2018). Our approach to modelling longitudinal mediation processes was informed by Selig and Preacher (2009) and a two‐timepoint adaptation thereof by Yip et al. (2024). For all models, we applied maximum likelihood estimation with robust standard errors to assess model fit (RMSEA, CFI, TFI, SRMR; Hu & Bentler, 1999). Given our relatively small sample sizes, especially in the T3–T1 samples, RMSEA values approaching 0.08 and SRMR values close to or below 0.09 were considered acceptable. For TLI and CFI, values close to or exceeding 0.95 indicated good fit. Indirect effects were tested utilizing a bias‐corrected bootstrap procedure (10,000 replications) to estimate 95% confidence intervals (MacKinnon et al., 2004).

## STUDY 1: CLIMATE CAMP (2019)

Data for Study 1 were collected among the estimated 1000 participants of a climate camp in Germany in August 2019. These camps are approximately week‐long gatherings of the climate justice movement (Müller, 2023). The camp included an educational programme with interactive workshops and panel discussions on topics of socio‐ecological change. However, climate camps also serve as a space to practice and enact visions of a socially just and ecologically sustainable society within the context of the camp (Frenzel, 2014; Russell et al., 2017).

### Method

#### Participants and procedure

Participants were invited to take part in the pre‐survey (T1) before or upon their arrival at the camp (informed consent was obtained). Those who participated in the pre‐survey at the camp were able to choose from an online and paper‐pencil questionnaire. All other surveys were administered online. Questionnaires were provided in German and English (88% of the respondents chose the German questionnaire at T1). In the final samples, we included participants who answered all study‐relevant items in the pre‐ and the post‐survey (T2–T1) and/or in the pre‐ and follow‐up survey (T3–T1). The T2–T1 sample contained 169 participants (68% identified as female, *M*_age_ = 27, *SD*_age_ = 7.40) and the T3–T1 sample 97 participants (64% identified as female, *M*_age_ = 28, *SD*_age_ = 8.89). The sample had a strong left leaning political orientation, scoring an average of 1.77 (*SD* = 0.98) on the widely used 11‐point left–right schema (Fuchs & Klingemann, 1990). For comparison, the average score for the German population was 5.37 (*SD* = 1.84) at the same time (Forschungsgruppe Wahlen, 2020).

#### Measures

All variables were measured on 7‐point Likert‐type scales (1 = *strongly disagree*, 7 = *strongly agree*; also see Supplement 1 for a list of all items). The factor loadings of all latent constructs were significant (see Table A1 in Appendix for factor loadings, means, standard deviations, and alpha values for all constructs at times 1, 2, and 3 for the entire sample). We applied item parcelling averaging the two most highly correlated items of our measures of collective action intentions (items 3 and 4) and collective efficacy beliefs (items 3 and 4) into a single indicator.

*Collective action intentions*. Participants of climate camps are often actively involved in the climate justice movement. To reduce the risk of ceiling effects, we used a measure with high item difficulty referring to participants' willingness to engage high‐cost activist behaviours for climate justice: (1) being part of an action group even if it takes up a large part of one's free time, (2) taking part in demonstrations, even if it is very costly, (3) breaking laws and living with the legal consequences, and (4) participating in civil disobedience, even if it might have negative consequences for one personally.

*Socio‐ecological cognitive alternatives*. As there were no validated scales on cognitive alternatives in 2019, we developed our own measure: (1) I have a clear picture of how a socially and ecologically just society could look like, (2) It is clear to me what is necessary to make society socially and ecologically just, (3) I can well imagine what it is like to live in a society based on solidarity. These items are conceptually similar to the scale developed by Wright et al. (2020), which was published in the course of the following year.

*Collective efficacy beliefs* were measured with four items adapted from Jugert et al. (2016): As the climate justice movement, we can collectively (1) plan and implement effective actions for climate justice, (2) change society in a relevant positive way, (3) make an important contribution to fighting the climate crisis within the next 10 years, and (4) make society more socially and ecologically just within the next 10 years.

The measurement of *movement identification* was based on group‐level self‐investment (Leach et al., 2008): (1) strongly identifying, (2) feeling a strong bond, and (3) being happy to be part of the climate justice movement.

### Results and discussion

We constructed each latent variable individually and tested for measurement invariance across pre‐, post‐, and follow‐up surveys. All variables were constrained to have strict measurement invariance to ensure comparability across time points (see Supplement 2). We built separate measurement models for pre–post changes (T2–T1) and changes between pre and follow‐up (T3–T1). All measurement models yielded good or acceptable fit (see Table A2 in Appendix).

#### Change score correlations

Table 1 presents the correlations between the latent change scores for all study variables. For both T2–T1 and T3–T1, change in cognitive alternatives was positively correlated with change in collective efficacy and with change in movement identification. In turn, changes in these two variables were positively correlated with change in collective action intentions. Notably, there were no significant bivariate correlations between change in cognitive alternatives and change in collective action intentions.

**TABLE 1:** Correlation of latent change scores (LCSs) in Study 1.

		Δ T2–T1				Δ T3–T1			
		1	2	3	4	1	2	3	4
1 Δ	Collective action intention	1				1			
2 Δ	Cognitive alternatives	0.03	1			0.20	1		
3 Δ	Collective efficacy	0.35*	0.35*	1		0.37**	0.47*	1	
4 Δ	Movement Identification	0.54***	0.42***	0.38**	1	0.65***	0.36*	0.47***	1

* < 0.05.

** < 0.01.

*** < 0.001.

#### Simple mediation models

**FIGURE 2:**
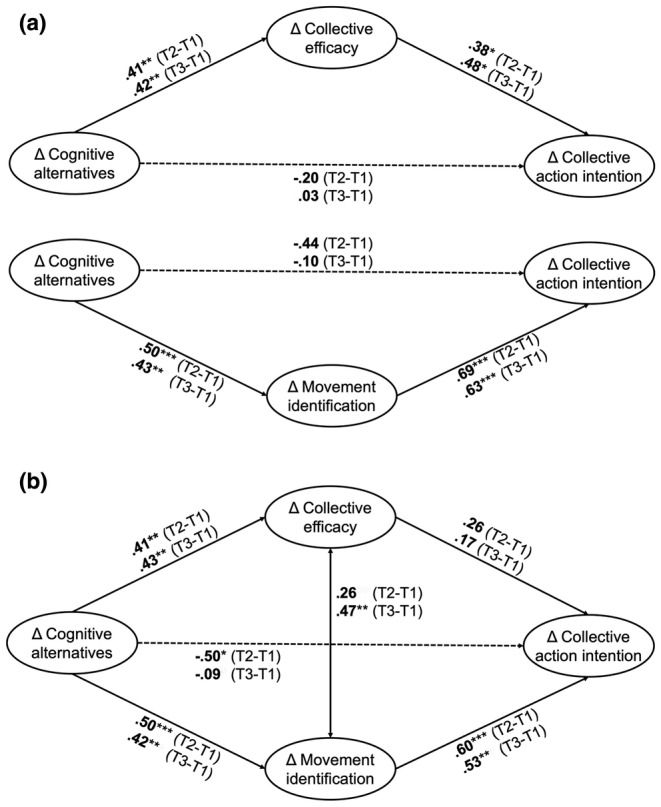
Latent change score mediation models in Study 1. (a) Simple mediation models for separate analysis of each path. (b) Parallel mediation model for analysis of both paths together. Presented values are standardized coefficients. The display of model paths and results has been reduced to the paths relevant for this research. For an overview of the parallel mediation model including all modelled regression and covariation paths, see Figure A1 in Appendix.

First, we built simple mediation models to test the efficacy and the identification pathway separately (see Figure 2a and Supplement 3 for detailed regression results). The results of the simple mediation analyses supported our assumptions: For collective efficacy beliefs, we found significant indirect effects for T2–T1 and T3–T1, indicating that the relationship between change in cognitive alternatives and change in collective action intentions was mediated by change in collective efficacy beliefs (T2–T1: b* = .16, 95% CI [0.01, 0.45]; T3–T1: b* = .20, 95% CI [0.01, 0.86]). Similarly, change in movement identification was found to mediate the association between change in cognitive alternatives and change in collective action intentions (T2–T1: b* = .34, 95% CI [0.10, 0.69]; T3–T1: b* = .27, 95% CI [0.03, 0.78]).

#### Parallel mediation models

Next, we integrated the efficacy path and the identification path into parallel mediation models for T2–T1 and T3–T1 (see Figure 2b and Supplement 3 for detailed regression results). The results partly support our assumptions: The indirect effect through change in collective efficacy beliefs was no longer significant when including change in movement identification as a parallel mediator (T2–T1: b* = 0.11, 95% CI [−0.02, 0.38]; T3–T1: b* = 0.07, 95% CI [−0.18, 1.05]). In contrast, change in movement identification remained a significant mediator of the relationship between change in cognitive alternatives and change in collective action intentions (T2–T1: b* = 0.30, 95% CI [0.08, 0.67]; T3–T1: b* = 0.22, 95% CI [0.006, 0.66]). As a post‐hoc power analysis, we conducted a Monte Carlo simulation with pwrSEM (Wang & Rhemtulla, 2021) using 10,000 replications for the T2–T1 and T3–T1 parallel mediation models. Results indicated that our tests for indirect effects were partly underpowered, particularly in the smaller T3–T1 sample (achieved power for indirect effects T2–T1: collective efficacy = 13%, movement identification = 82%; T3–T1: collective efficacy = 5%, movement identification = 33%; see Supplement 4 for detailed results).

#### Discussion

Study 1 provides initial evidence for the efficacy and identification pathways from imagination to activism. Simple mediation analyses showed that changes in the accessibility of cognitive alternatives positively predicted changes in collective action intentions through changes in collective efficacy beliefs and changes in identification with the climate movement. Put differently, increases in cognitive alternatives predicted higher levels of collective efficacy beliefs or movement identification, which subsequently predicted stronger collective action intentions. However, when including changes in collective efficacy beliefs and movement identification as parallel mediators, the link between collective efficacy beliefs and collective action intentions dropped to non‐significance. This may suggest that, although efficacy beliefs contribute to the link between imagination and activism, the process through politicized identification is more robust. Results of post‐hoc power analysis also demonstrated that achieved power was lower for the indirect effect through collective efficacy beliefs than for the indirect effect through movement identification. In other words, our sample might have been too small to reliably detect collective efficacy as a parallel pathway. Notably, the inclusion of both mediators in the parallel mediation model revealed an unexpected negative direct effect of changes in cognitive alternatives on changes in action intentions for T2–T1. As the bivariate correlation between changes in cognitive alternatives and collective action intentions was not negative before, this might point to a suppression effect (see Rucker et al., 2011). This means an increase in cognitive alternatives might have had a negative effect on collective action intentions once the positive effects of collective efficacy and movement identification were accounted for (also see General discussion). To test the robustness of our findings, Study 2 aimed to replicate the current results in a different field context, with a bigger sample, for a slightly different reference group, and over a longer time period.

## STUDY 2: ‘FUTURE FOR ALL’ ONLINE CONFERENCE (2020)

Study 2 was conducted as part of the evaluation of an online conference on visions of a just and ecologically sustainable society. The five‐day ‘Future for All’ conference took place in August 2020 was attended by approximately 1500 participants and, like the climate camp, included interactive workshops and panel discussions. However, the conference addressed a broader audience of actors working for socio‐ecological change in different areas of society, especially actors from civil society (e.g., social movement organizations, NGOs, and foundations), education, and the sciences. Also, as an additional mediator, we explored *participative efficacy*, the belief that one's own action will make a difference in achieving collective goals (Van Zomeren et al., 2013). Increased cognitive alternatives might not only lift collective efficacy beliefs, but also raise the perceived importance of one's own contribution to the collective pursuit of social change. Extending previous research, Study 2 aimed at exploring which kinds of efficacy beliefs mediate the path from imagination to activism.

### Method

#### Participants and procedure

Conference participants were invited to take part in the pre (T1), post (T2), and follow‐up (T3) online surveys (informed consent was obtained). As in Study 1, questionnaires were provided in German and English (only 4 respondents [1%] chose the English questionnaire at T1). Again, we only included participants who answered to all study‐relevant items in the pre‐ and post‐surveys (T2–T1) and/or in the pre and follow‐up surveys (T3–T1). The T2–T1 sample contained 273 participants (62% identified as female, *M*_age_ = 35, *SD*_age_ = 11.95) and the T3–T1 sample 186 participants (60% identified as female, *M*_age_ = 36, *SD*_age_ = 12.46). As in Study 1, participants had a strong left leaning political orientation (*M* = 1.74, *SD* = 1.09).

#### Measures

We used slightly different measures than in Study 1 adapted to the context of socio‐ecological transformation, the topic of the conference. All variables were measured with three items on 7‐point Likert‐type scales (1 = *absolutely disagree*, 7 = *absolutely agree*; also see Supplement 1 for a list of all items). The factor loadings of all latent constructs were significant (see Table A3 in Appendix for factor loadings, means, standard deviations, and alpha values for all constructs at times 1, 2, and 3 for the entire sample).

For *collective action intentions*, participants were again asked to indicate their willingness to engage in high‐cost activist behaviours, this time with regard to socio‐ecological justice: (1) participate in actions of civil disobedience, even if this has negative consequence for them personally; (2) do poorly or completely unpaid work for a political organization, even if this makes life considerably more strenuous and stressful; and (3) take part in demonstrations on a regular basis, even if this leaves little free time.

*Socio‐ecological cognitive alternatives*. As there were no published scales on cognitive alternatives at the time of this study, we used a self‐developed measure similar to Study 1: (1) I have a concrete vision of how a socially and ecologically just society could look like. (2) It is very clear for me how life in a society based on global and local solidarity would be like. (3) I find it difficult to clearly imagine a socially and ecologically just society (reversed).

We assessed *collective and participative efficacy beliefs* each with three items adapted from Hamann et al. (2021). Collective efficacy was measured by asking to which extent participants agree that together as social–ecological movements, we can (1) push forward the fundamental transformation of our society, (2) contribute crucially to make the whole world significantly more just, and (3) realize a society based on global and local solidarity. The three items for *participative efficacy* were similar but tapped into the respondents' perceived personal ability to contribute to these collective efforts (e.g., ‘I, as an individual, can contribute substantially so that we as social–ecological movements can push forward the fundamental transformation of our society’).

Finally, the measurement of *movement identification* was again based on group‐level self‐investment (Leach et al., 2008): (1) I feel a very strong bond with movements for social–ecological justice. (2) It is very fulfilling for me to be part of movements for social–ecological justice. (3) Belonging to movements for social–ecological justice is one of the most important parts of my identity.

### Results and discussion

We followed the same analysis strategy as in Study 1. All measurement models for both pre–post changes (T2–T1) and changes between pre and follow‐up (T3–T1) yielded good fit (see Table A4 in Appendix).

#### Change score correlations

For T2–T1, change in cognitive alternatives was positively correlated with change in collective efficacy and movement identification, but not participative efficacy (see Table 2). This replicates the findings from Study 1. For T3–T1, the associations between change in cognitive alternatives and collective efficacy, or movement identification were not significant. As in Study 1, we found no significant bivariate correlations between change in cognitive alternatives and change in collective action intentions.

**TABLE 2:** Correlation of latent change scores (LCSs) in Study 2.

		Δ T2–T1					Δ T3–T1				
		1	2	3	4	5	1	2	3	4	5
1 Δ	Collective action intention	1					1				
2 Δ	Cognitive alternatives	.20	1				−0.16	1			
3 Δ	Collective efficacy	0.40***	0.38***	1			0.28*	0.15	1		
4 Δ	Participative efficacy	0.20*	0.22	0.57***	1		0.18	0.21	0.53***	1	
5 Δ	Movement Identification	0.55*	0.36**	0.46***	0.27**	1	0.49***	0.16	0.33**	0.31**	1

* < 0.05.

** < 0.01.

*** < 0.001.

#### Simple mediation models

**FIGURE 3:**
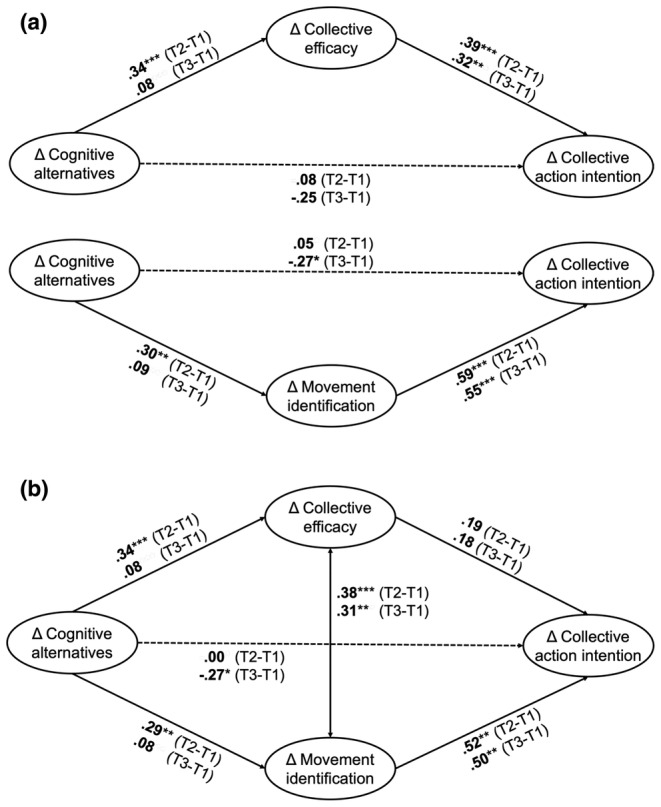
Latent change score mediation models in Study 2. (a) Simple mediation models for separate analysis of each path. (b) Parallel mediation model for analysis of both paths together. Presented values are standardized coefficients. The display of model paths and results has been reduced to the paths relevant for this research. For an overview of the parallel mediation model including all modelled regression and covariation paths, see Figure A1 in Appendix.

Like in Study 1, we first ran separate mediation models including change in collective efficacy beliefs or change in movement identification as single mediators (see Figure 3a and Supplement 3 for detailed regression results). Again, results showed that change in collective efficacy beliefs mediated the effect of change in cognitive alternatives on change in collective action intentions for T2–T1, b* = 0.13, 95% CI [0.07, 0.36]. Similarly, we found a positive indirect effect through change in movement identification for T2‐T1 changes, b* = 0.18, 95% CI [0.05, 0.49]. For T3–T1, the indirect effects through collective efficacy beliefs (b* = 0.03, 95% CI [−0.03, 0.13]) and through movement identification (b* = 0.05, 95% CI [−0.06, 0.20]) were not significant.

#### Parallel mediation models

When modelling changes in collective efficacy beliefs and movement identification as parallel mediators (see Figure 3b and Supplement 3 for detailed regression results), results showed a significant positive indirect effect through change in movement identification for T2–T1, b* = 0.19, 95% CI [0.06, 0.38]. In contrast, change in collective efficacy no longer mediated the effect of change in cognitive alternatives on change in collective action intentions for T2–T1 in the parallel mediation model, b* = 0.07, 95% CI [−0.02, 0.22]. This corroborates previous findings. For T3–T1, we found no significant indirect effects through collective efficacy beliefs (b* = .01, 95% CI [−0.01, 0.10]) or movement identification (b* = 0.04, 95% CI [−0.06, 0.18]) in the parallel mediation model. Results of post‐hoc Monte Carlo simulations revealed that our tests for indirect effects in the parallel mediation models were – by and large – underpowered (T2–T1: collective efficacy = 23%, movement identification = 68%; T3–T1: collective efficacy = <1%, movement identification = 6%, see Supplement 4 for detailed results).

#### Participative efficacy as additional efficacy‐related mediator?

We built an exploratory model including changes in participative efficacy and collective efficacy beliefs as two parallel efficacy‐related mediators for T2–T1 (see Supplement 3 for detailed regression results). Change in collective efficacy beliefs remained a significant mediator of the relationship between changes in cognitive alternatives and collective action intentions, b* = .19 [95% CI 0.06, 0.38]. In contrast, we found no indirect effect through change in participative efficacy, b* = 0.00 [95% CI‐0.06, 0.06].

#### Discussion

Results of Study 2 provided additional, but not full, support for the efficacy and identification pathways from imagination to activism. Specifically, collective efficacy beliefs and movement identification emerged as significant mediators of the effect of cognitive alternatives on collective action intentions in separate (simple) mediation models for pre–post changes (but not for changes between pre and follow‐up). Furthermore, changes in collective efficacy beliefs, yet not in participative efficacy, mediated the relationship between changes in cognitive alternatives and collective action intentions. This indicates that the mental accessibility of societal alternatives is linked to collective action through perceptions of collective, but not personal control. When including collective efficacy beliefs and movement identification as parallel mediators in the analysis, the positive indirect effect through movement identification remained significant for T2–T1. In contrast, the positive indirect effect through collective efficacy beliefs dropped to non‐significance. However, results of post‐hoc power analysis revealed that the indirect effect through collective efficacy was substantially underpowered. To increase power, we reran the parallel mediation model with a pooled sample of both Study 1 and 2 (see Pooled analysis below). Moreover, for T3–T1, the inclusion of collective efficacy beliefs and movement identification as parallel mediators resulted in a negative direct effect of change in cognitive alternatives on change in collective action intentions. Similar to the T2–T1 results in Study 1, this may again suggest a suppression effect (also see General discussion).

Our findings from T2–T1 are in line with the results of Study 1. However, unlike in the first study we did not find significant mediation effects for collective efficacy and movement identification for T3–T1. Specifically, changes in cognitive alternatives did not predict changes in either of the two proposed mediators. In contrast, changes in collective efficacy and movement identification both remained similarly strong predictors of changes in collective action intentions. We can only speculate about the reasons for this inconsistency. One explanation might be the timing of the follow‐up measurement (T3) in Study 2. The follow‐up took place in September 2021, amid the second year of the corona pandemic as well as during the lead‐up to the German federal elections. In this situation, the effects of a change in socio‐ecological cognitive alternatives might have been cancelled out by the limited perceived agency of social movements in implementing such change (also see General discussion).

## POOLED ANALYSIS

The parallel mediation models in Study 1 and 2 including both the efficacy and the identification path were partly underpowered, particularly for the test of collective efficacy beliefs as a unique mediator. To increase statistical power, we decided to pool the data from Study 1 and Study 2. We only merged T2–T1 data, because it was collected directly before and after the week‐long events in both studies and, therefore, ensured a consistent measurement interval across the pooled datasets.

### Method

We adopted a manifest approach to LCS modelling with a single mean‐summarized indicator for each timepoint (for examples of single‐ and multiple‐indicator LCS models, see Kievit et al., 2018). All measures used in the pooled analysis had acceptable internal consistency (see Tables A2 and A3 in Appendix) and were standardized to ensure compatibility across the pooled datasets. Data pooling resulted in a total sample of 442 participants.

### Results and discussion

In line with the results of Study 1 and Study 2, change in cognitive alternatives was positively correlated with change in collective efficacy and movement identification. There was no significant bivariate correlation between change in cognitive alternatives and change in collective action intentions (see Table 3).

**TABLE 3:** Correlation of latent change scores (LCSs) in the pooled data.

		Δ T2–T1			
		1	2	3	4
1 Δ	Collective action intention	1			
2 Δ	Cognitive alternatives	0.11	1		
3 Δ	Collective efficacy	0.36***	0.27***	1	
4 Δ	Movement Identification	0.36***	0.25***	0.34***	1

* < 0.05, ** < 0.01.

*** < 0.001.

**FIGURE 4:**
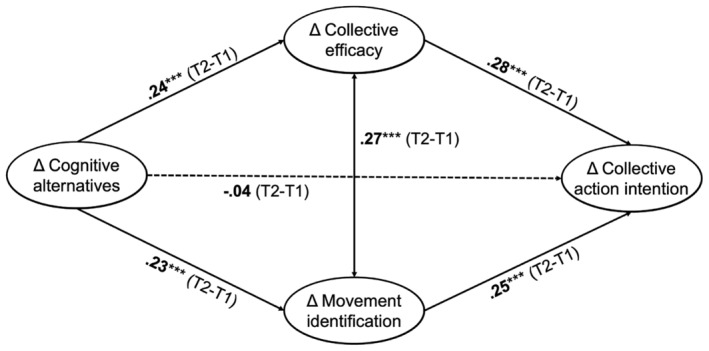
Latent change score mediation model of the pooled analysis. Presented values are standardized coefficients. The display of model paths and results has been reduced to the paths relevant for this research. For an overview of the parallel mediation model including all modelled regression and covariation paths, see Figure A1 in Appendix.

We ran a parallel mediation model including change in collective efficacy beliefs and change in movement identification as parallel mediators (see Figure 4 and Supplement 3 for detailed regression results). Model fit was good: *χ* ^2^ = 27.33 (7); RMSEA: 0.08^2^ (90% CI [0.04–0.12]); CFI: 0.99; TLI: 0.96; SRMR: 0.05. In line with our assumptions, we found significant positive indirect effects for collective efficacy beliefs, b* = 0.06, 95% CI [0.03, 0.11], as well as for movement identification, b* = 0.05, 95% CI [0.06, 0.11].^3^ A post‐hoc Monte Carlo simulation confirmed that we had sufficient power to detect both indirect effects (collective efficacy = 100%, movement identification = 100%, see Supplement 4 for detailed results). The analysis of the pooled data from both studies thus confirmed collective efficacy and movement identification as unique (positive) pathways from imagination to activism.

## GENERAL DISCUSSION

As human beings, our imagination enables us to act on the basis of what does not (yet) exist and change our social reality towards these imagined societal possibilities (Bloch, 1985; Zittoun & Gillespie, 2016). The present research contributes to the understanding of the psychological processes underpinning this link between imagined possibility and collective action. Drawing on social identity theory (Tajfel & Turner, 1979) and corresponding research on collective (environmental) action (e.g., Fritsche et al., 2018; Fritsche & Masson, 2021; Van Zomeren et al., 2008), we proposed that collective efficacy beliefs and politicized identification would form two pathways from imagination to activism. Our two field settings provided a unique opportunity to examine these processes over time and under conditions of high ecological validity.

### Key findings

The current research provides initial evidence that being able to imagine social change is accompanied by a sense of collective efficacy and identification with social movements, which, in turn, can motivate people to engage in collective change‐directed action. The results confirm these pathways from imagination to activism when predicting changes immediately after each of the two week‐long events (Studies 1 and 2, pooled analysis) as well as 5 months later (Study 1) but not 13 months after the event investigated in Study 2.

The lack of long‐term indirect effects in Study 2 might be due to externally induced changes in the perceived agency of socio‐ecological movements during the measurement period. While Study 1 (including the follow‐up measurement) took place before the COVID‐19 pandemic in a time of record‐breaking climate protest initiated by the climate strike movement (Sommer & Haunss, 2020), such activities had markedly dropped at the time of Study 2, especially at its follow‐up measurement in September 2021. This measurement took place in the second year of the pandemic and in the immediate lead‐up to the German federal elections. By then, the impact of socio‐ecological movements had stagnated for over a year (de Moor et al., 2021; Mayer et al., 2021; Rucht & Rink, 2020). Furthermore, instead of a dynamic climate movement exerting pressure on politics, the political discourse before the elections was once again dominated by the usual actors of parliamentary politics in Germany (Ackermann et al., 2023; Faas & Klingelhöfer, 2022; Schmitt‐Beck, 2021). This situation was not necessarily characterized by stability. In fact, pre‐election polls indicated a shift towards a less conservative and more liberal government (wahlrecht.de, 2021). However, this development was not brought about by socio‐ecological movements. On the contrary, the green and the left party, which are the closest to representing the interests of progressive social movements in Germany, were declining in the polls in the months leading up to the election (Forsa, 2024). In this situation, changes in the accessibility of socio‐ecological cognitive alternatives were likely contrasted by the stagnating capability of social movements to implement such change. This situationally induced salience of the limited collective agency of socio‐ecological movements might have cancelled out the effect of cognitive alternatives on collective efficacy beliefs and movement identification. Or, in other words, it might have blocked the two pathways from imagination to activism.

Notably, the results showed no significant positive correlation between changes in cognitive alternatives and collective action intentions in both studies. In contrast, we found negative *direct* effects in two of the parallel mediation models. These presumed suppression effects (see Rucker et al., 2011) indicate that an increase in cognitive alternatives might have a negative effect on collective action intentions once the positive effects of collective efficacy and movement identification are controlled for. An explanation for this negative effect might be the perceived contrast between imagined societal alternative and the appraisal of the current society. While an increase in cognitive alternatives can trigger collective action by creating a sense of collective efficacy and movement identification, at the same time, it might also make people aware of how much their desired society deviates from the current development. The increased salience of such is‐ought discrepancies might de‐motivate people to act, at least when parallel increases in collective efficacy and movement identification are taken out of the equation.

### Implications and future directions

The ability to imagine a desirable society can motivate social change activism by increasing the perception of collective agency: perceiving oneself as part of a group (i.e., movement identification) that has the capability to change society (i.e., collective efficacy). This confirms earlier laboratory findings that (ecological) visioning tasks increase people's (pro‐environmental) social change motivation (Bosone et al., 2023; Daysh et al., 2024; Fernando et al., 2018, 2020). Meanwhile, the present results show why this takes place. Regarding the *efficacy path*, we theorized that the ability to imagine socio‐ecological change increases people's perceived possibility of and self‐efficacy regarding, societal change. Indeed, we conceptually replicated in ecologically valid field settings and parallel to laboratory findings (Bosone et al., 2023) that increased cognitive alternatives predict an increase in collective efficacy beliefs. This increase in collective efficacy beliefs mediated the positive relationship between changes in cognitive alternatives and collective action intentions. With regard to the *identification path*, we reasoned that being able to envision social change increases one's identification with those social movements, whose norms align with one's imagination of a better society. In turn, this heightened politicized identification boosts one's motivation to engage in change‐directed action. Our results support this hypothesis and are consistent with findings from Wright et al. (2022) whose cross‐sectional analyses showed that the relationship between environmental cognitive alternatives and pro‐environmental action is mediated by the identification with environmental activists. We extend these cross‐sectional findings by demonstrating a similar mediation process longitudinally.

#### Implications for the link between the mental accessibility of societal alternatives and social change motivation

The absence of a clear direct relationship between changes in cognitive alternatives and collective action intentions underscores the crucial role of the investigated mediators. For the participants in our two studies, increases in collective efficacy and especially politicized identification appear to be inevitable intermediate steps on the path from imagination to activism. These results are at odds with cross‐sectional findings showing direct links between environmental cognitive alternatives and corresponding action intentions (Wright et al., 2020, 2022), as well as direct effects of imagination tasks on social change motivation (Bosone et al., 2023; Fernando et al., 2018, 2020). Apart from methodological differences such as operationalizations and research designs (longitudinal vs. cross‐sectional vs. experimental), one reason for this inconsistency may be that, unlike previous studies, our research focused on high‐cost activist behaviour in a sample of highly politicized individuals. There may be a general effect of the accessibility of societal alternatives on social change motivation. However, this direct relationship may be weaker for those who are already politically active and their propensity to make further personal sacrifices for their activism. In this case, it seems that an increase in cognitive alternatives needs to elicit a sense of being part of an agentic movement in order to motivate collective action. Future research should put these considerations to the test. For instance, by experimentally manipulating the mediators of the proposed pathways from imagination to activism, or by longitudinally investigating whether similar processes occur in samples more representative of the general population.

#### Implications for efficacy beliefs and related constructs

Preliminary evidence indicating that efficacy beliefs may play a mediating role between imagination and activism came from Fernando et al. (2020). They showed that the effect of imagining a green sufficiency society (vs. a high‐tech abundance society) on social change motivation was mediated by *participative efficacy* beliefs, the belief that ‘Ordinary people can help realize this version of society’ (p. 7). However, we did not conceptually replicate an independent role of participative efficacy in mediating the effect of cognitive alternatives. Possibly, the effect of participative efficacy found by Fernando et al. (2020) might trace back to the specific content of the two visions that were compared in this study. On the contrary, our results indicate that the motivational power of having a vision is more about realizing the collective possibilities for change and less about one's individual ability to contribute to the collective change effort. Future research should continue to include both collective and participative efficacy beliefs to see whether this conclusion holds across different populations, especially less politicized samples.

In addition, recent studies have focused on *hope* as another mediator between imagination and change‐directed action. Both Daysh et al. (2024) and Badaan et al. (2022) showed that engaging with a societal vision led to increased hope, which in turn predicted collective action intentions. Furthermore, Troy et al. (2024) found that a positive message describing societal changes to address climate change increased hope when collective efficacy beliefs were high. Conversely, Cohen‐Chen and Van Zomeren et al. (2018) had previously demonstrated that collective efficacy beliefs predicted collective action only when hope was high. Although these studies used different operationalizations of hope, their findings have two general implications: first, hope may be another mediator of the effect of having a vision, and second, the effects of hope and efficacy beliefs may interact. It is plausible that both efficacy and hope are related to the experience and appraisal of (change) possibility. Future research should investigate the role of these affective and cognitive aspects of possibility perception for the link between imagination and action. A good starting point would be to adapt a consistent operationalization of hope and to clarify its dynamics with efficacy beliefs as well as possible joint effects on social change motivation.

#### Implications for social identity models of collective action

The findings presented in this paper also have implications for social identity models of collective action (Agostini & Van Zomeren, 2021; Fritsche et al., 2018; Thomas et al., 2011; Van Zomeren et al., 2008), which posit that collective efficacy and ingroup identification are key drivers of collective action (alongside injustice perceptions and ingroup norms). Our results suggest a possible extension of these models, as these collective action motives may share cognitive alternatives as a common predictor. Moreover, collective action models typically depart from negative collective states, such as social injustice (Van Zomeren et al., 2008) or collective environmental crises (Fritsche et al., 2018), which are thought to motivate collective action to alleviate these miseries. Our findings, however, indicate that positive collective states, like those envisioned as desired societal alternatives, can also drive collective action and are associated with similar processes. To broaden our understanding of these processes, future research should also include other key motives of collective action, especially perceived injustice and personal moral norms (Agostini & Van Zomeren, 2021; Barth et al., 2015; Van Zomeren et al., 2018). It is plausible that increasing the accessibility of societal alternatives raises awareness not only of collective possibilities for social change – *how it could be* – but also of the moral norms underlying these desired alternatives – *how it should be*.

Furthermore, our findings suggest that out of the two pathways tested in this study, movement identification may be the more stable pathway from imagination to activism. This is in line with findings from collective action research, which consistently showed that politicized identification is one of the strongest and most proximal predictors of collective action (Schmitt et al., 2019; Simon et al., 1998; Van Zomeren et al., 2008) and that the path from group efficacy to collective action is of smaller size (Thomas et al., 2011). These results have implications for the debate about whether group membership leads to the experience of efficacy (as in SIMCA, Van Zomeren et al., 2008) or, conversely, whether the experience of efficacy influences our identification as a group member (as in EMSICA, Thomas et al., 2011). In light of this debate, we would argue that our findings hint towards the latter direction of causality: having a vision motivates social change because it contributes to the construction of an agentic social self, that is, it increases collective efficacy beliefs and thereby identification with change‐oriented groups.

### Methodological limitations

Longitudinal studies do only provide limited inference about the causality of effects. Our theoretical rationale and the correlational pattern of the changes among our variables (see Tables 1, 2, 3) speak for the presented causal mediational model. Nonetheless, with an observational design, it is not possible to authoritatively adjudicate the causality of the efficacy and identification path. For instance, as an alternative model, increased identification with a social movement may have strengthened the accessibility of cognitive alternatives through highly involved group members being strongly exposed to alternative modes of thinking and acting which are practised and disseminated by the movement. Due to these interpretational ambiguities of longitudinal field research, the present research should be seen as complementing experimental findings on utopian thinking (Fernando et al., 2018, 2020) and the exposure to societal visions (Bosone et al., 2023; Daysh et al., 2024) as well as on the determinants of collective action (e.g., Fritsche et al., 2018; Van Zomeren et al., 2008). The main value of the current research is the application and replication of such findings in a setting with high ecological validity as well as the integrated analysis of both the collective efficacy and the identification path.

### Conclusion

Having a vision, being able to imagine alternatives to the status quo, has the potential to foster people's sense of agency by identifying as part of an efficacious social movement. It may even be a vantage point for social change activism. The processes linking imagination and activism are less about people's individual efficacy than about realizing the collective possibilities for social change and identifying with the groups enacting it. Such a sense of joint striving and efficacy is what might bridge subjective helplessness in the face of large‐scale social and environmental crises that often prevents people to act (Fritsche et al., 2018; Marder et al., 2023). From an applied perspective, research on the mental accessibility of societal alternatives can help us better understand how we, as societies, can move on from the status quo towards a socially and ecologically just alternative. At a theoretical level, it may be a step towards solving the puzzle of collective agency.

## AUTHOR CONTRIBUTIONS

**Julian Bleh:** Conceptualization; methodology; investigation; data curation; formal analysis; project administration; visualization; writing – original draft; writing – review and editing. **Torsten Masson:** Conceptualization; methodology; supervision; writing – original draft; writing – review and editing; project administration. **Sabrina Köhler:** Methodology; investigation. **Immo Fritsche:** Conceptualization; supervision; writing – review and editing; writing – original draft.

## Supporting information


Data S1.

## Data Availability

The authors confirm that the data supporting the findings of this study will be published within its supplementary materials: https://osf.io/gvw2y/.

## Footnotes

^1^
The focus of the current research lies on modelling paths between change scores. LCS models can also be used to investigate associations within a single time point and paths from variables at Time 1 to theoretically consequent changes in other variables (e.g. the effect of a variable at T1 on changes in another variable between T1 and T2). These paths were included in our models for fit purposes and to control for baseline levels. However, we did not have specific hypotheses about these associations and will not report them in this paper (for more details, see Figure 2 in Appendix and the supplementary material).

^2^
The RMSEA value is reported here but was not included in the assessment of model fit, because it tends to be inflated for small degrees of freedom (see Kenny et al., 2015).

^3^
In general, the *b**‐weights of all regression and indirect effects were smaller than in the previous analyses. These differences are probably a consequence of using a manifest (single‐indicator) approach rather than error‐free latent variables for each time point.

## APPENDIX A

### A.1. Study 1: Climate camp

**TABLE A1:** Descriptive statistics for constructs in Study 1. Factor loadings are standardized. Means, standard deviations, and alpha values were calculated before item parcelling of the collective action intentions and collective efficacy indicators.

Latent variable	Time				Factor loadings per indicator		
		M	SD	α	i1	i2	i3
Collective action intentions	T1	4.89	1.26	.80	0.75	0.81	0.58
	T2	5.12	1.21	.80	0.81	0.76	0.57
	T3	4.99	1.29	.85	0.90	0.80	0.58
Cognitive alternatives	T1	5.24	1.12	.79	0.90	0.84	0.53
	T2	5.59	0.91	.76	0.85	0.78	0.54
	T3	5.34	1.05	.76	0.85	0.86	0.46
Collective efficacy	T1	5.54	0.85	.84	0.69	0.82	0.82
	T2	5.51	0.91	.88	0.80	0.82	0.85
	T3	5.49	0.88	.85	0.62	0.76	0.92
Movement identification	T1	5.53	1.19	.88	0.90	0.84	0.78
	T2	5.63	1.14	.89	0.88	0.85	0.84
	T3	5.50	1.22	.90	0.85	0.95	0.79

**TABLE A2:** Model fit indices for Study 1.

Analysis	Timeframe	Fit indices				
		*χ* ^2^ (df)	RMSEA (90% CI)	CFI	TLI	SRMR
Measurement model	T2–T1	285.88 (195)	0.05 (0.03, 0.06)	0.97	0.96	0.06
	T3–T1	278.51 (195)	0.06 (0.04, 0.08)	0.94	0.94	0.07
LCS correlation	T2–T1	380.40 (252)	0.05 (0.04, 0.06)	0.96	0.95	0.07
	T3–T1	380.98 (252)	0.07 (0.05, 0.09)	0.92	0.91	0.09
Efficacy path (simple mediation)	T2–T1	251.58 (135)	0.07 (0.05, 0.08)	0.94	0.93	0.07
	T3–T1	194.22 (135)	0.07 (0.04, 0.09)	0.94	0.93	0.08
Identification path (simple mediation)	T2–T1	201.65 (135)	0.05 (0.03, 0.07)	0.97	0.97	0.08
	T3–T1	173.53 (135)	0.05 (0.02, 0.07)	0.97	0.97	0.08
Both paths (parallel mediation)	T2–T1	375.08 (247)	0.05 (0.04, 0.06)	0.96	0.95	0.07
	T3–T1	366.13 (247)	0.07 (0.05, 0.08)	0.92	0.92	0.08

### A.2. Study 2: Online conference

**TABLE A3:** Descriptive statistics for constructs in Study 2. Factor loadings are standardized.

Latent variable	Time				Factor loadings per indicator		
		M	SD	α	i1	i2	i3
Collective action intentions	T1	4.80	1.08	.58	0.83	0.40	0.51
	T2	4.84	1.13	.70	0.77	0.58	0.66
	T3	4.73	1.12	.66	0.72	0.61	0.55
Cognitive alternatives	T1	4.71	1.59	.82	0.73	0.87	0.74
	T2	4.78	1.56	.82	0.86	0.79	0.70
	T3	4.82	1.56	.82	0.78	0.90	0.69
Collective efficacy	T1	5.38	1.11	.89	0.86	0.85	0.84
	T2	5.39	1.12	.91	0.93	0.85	0.84
	T3	5.26	1.00	.91	0.92	0.85	0.85
Participative efficacy	T1	4.61	1.33	.93	0.92	0.89	0.89
	T2	4.66	1.25	.91	0.91	0.84	0.88
	T3	4.50	1.27	.89	0.87	0.82	0.87
Movement identification	T1	4.99	1.29	.81	0.80	0.71	0.79
	T2	5.23	1.22	.86	0.83	0.80	0.83
	T3	5.17	1.22	.84	0.80	0.82	0.80

**TABLE A4:** Model fit indices for Study 2.

Analysis	Timeframe	Fit indices				
		*χ* ^2^ (df)	RMSEA (90% CI)	CFI	TLI	SRMR
Measurement model	T2–T1	648.02 (380)	0.05 (0.04, 0.05)	0.96	0.96	0.05
	T3–T1	576.42 (380)	0.05 (0.04, 0.06)	0.95	0.94	0.05
LCS correlation	T2–T1	682.64 (400)	0.05 (0.04, 0.05)	0.96	0.96	0.07
	T3–T1	598.36 (400)	0.05 (0.04, 0.06)	0.95	0.94	0.06
Efficacy path (simple mediation)	T2–T1	253.22 (135)	0.05 (0.04, 0.06)	0.97	0.96	0.06
	T3–T1	188.30 (135)	0.04 (0.02, 0.06)	0.97	0.97	0.06
Identification path (simple mediation)	T2–T1	268.91 (135)	0.06 (0.05, 0.07)	0.96	0.95	0.07
	T3–T1	185.92 (135)	0.04 (0.02, 0.06)	0.97	0.97	0.06
Both paths (parallel mediation)	T2–T1	437.66 (247)	0.05 (0.04, 0.06)	0.96	0.96	0.06
	T3–T1	377.32 (247)	0.05 (0.04, 0.06)	0.95	0.95	0.06
Additional analysis (participative & collective efficacy)	T2–T1	451.42 (247)	0.05 (0.04, 0.06)	0.96	0.96	0.06
	T3–T1	367.56 (247)	0.05 (0.03, 0.06)	0.96	0.96	0.06
