# Supplementary material for: From imagination to activism: Cognitive alternatives motivate commitment to activism through identification with social movements and collective efficacy
Source: Br J Soc Psychol. 2024 Nov 15;64(1):e12811. doi: 10.1111/bjso.12811 (PMC11590046; doi:10.1111/bjso.12811)
Supplement: Supplementary file 1 — Data S1. [file BJSO-64-0-s001.zip › Supp 4 power analyses/pwrSEM_MILCS_PM_Study2_T3-T1_N=186.pdf]

pwrSEM v0.1.2

Power Analysis for Parameter Estimation in Structural Equation Modeling

If you find this app useful, please cite: Wang, Y. A., & Rhemtulla, M. (in press (https://psyarxiv.com/pj67b)). Power analysis for parameter estimation in structural equation modeling: A discussion and tutorial. *Advances in Methods and Practices in Psychological Science.*

How to Use This App

Step 1. Specify Model. Enter your analysis model using lavaan syntax. Examples of formula types that define a structural equation model include (more information here (http://lavaan.ugent.be/tutorial/syntax1.html)):

- =~ "is measured by"
- ~ "is regressed on"
- ~~ "is correlated with"

Click "Set Model" to set the analysis model and continue to Step 2.

Step 2. Visualize. Ensure that the visualized model looks right, then click "Proceed" to continue to Step 3.

Step 3. Set Parameter Values. Fill in the "Value" column with the population value for each parameter, then check the boxes in the "Effect" column for the parameters you would like to detect. Click "Confirm Parameter Values" to continue to Step 4.

Step 4. Estimate Power. Set your sample size and number of simulations, then click

1. Specify Model 2. Visualize 3. Set Parameter Values 4. Estimate Power Help Resources

Set your sample size

186

Set your alpha level

0,05

Set seed for simulations

42

Set number of simulations

100 1,000 10,000

100 1,100 2,100 3,100 4,100 5,100 6,100 7,100 8,100 9,100 10,000

We recommend starting with a low number of simulations (e.g., 100) to get a rough estimate of power before confirming it with a higher number of simulations (e.g., 1000). The larger the number, the longer simulations will take.

Estimate Power via Simulations

Parameter	Value	Median	Power	Power (All Cases)
dCAI ~ dCOLL_EFF	0.12	0.12	0.33	0.33
dCOLL_EFF ~ dCOGN_ALT	0.11	0.12	0.17	0.17

"Estimate Power via Simulations" to run your power analysis.

Parameter	Value	Median	Power	Power (All Cases)
dCAI ~ dMOV_ID	0.40	0.39	0.96	0.96
dMOV_ID ~ dCOGN_ALT	0.09	0.10	0.14	0.14
dCAI ~ dCOGN_ALT	-0.25	-0.25	0.57	0.57
P1_indirect := a1_d*b1_d	0.01	0.01	0.00	0.00
P2_indirect := a2_d*b2_d	0.04	0.04	0.06	0.06
total := c_d+(a1_d*b1_d)+(a2_d*b2_d)#estimatetotaleffect	-0.20	-0.18	0.35	0.35

Convergence rate is 1. Value is the population parameter value as set in Step 3. Median is the median of simulated estimates of a parameter. Power is estimated from all simulations with converged models. Power (All Cases) is estimated from all simulations, including those with non-converged models (which had no parameter estimates and were counted as failure to reject the null).

Select parameter to display histograms

P1_indirect := a1_d*b1_d ▾

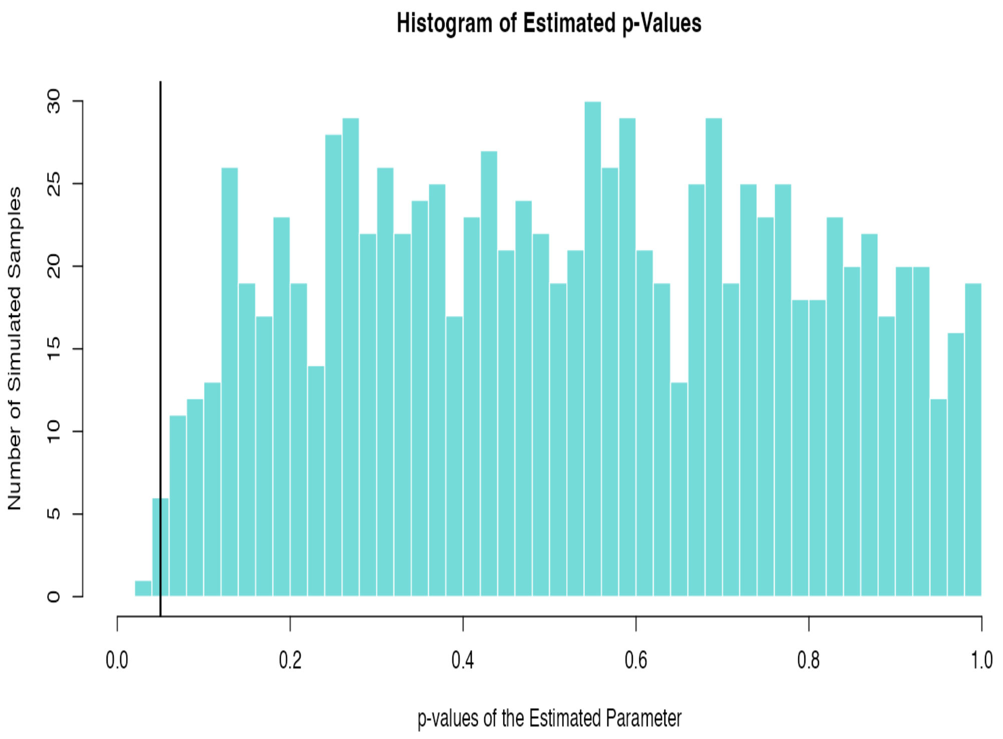

Vertical solid line indicates alpha level.

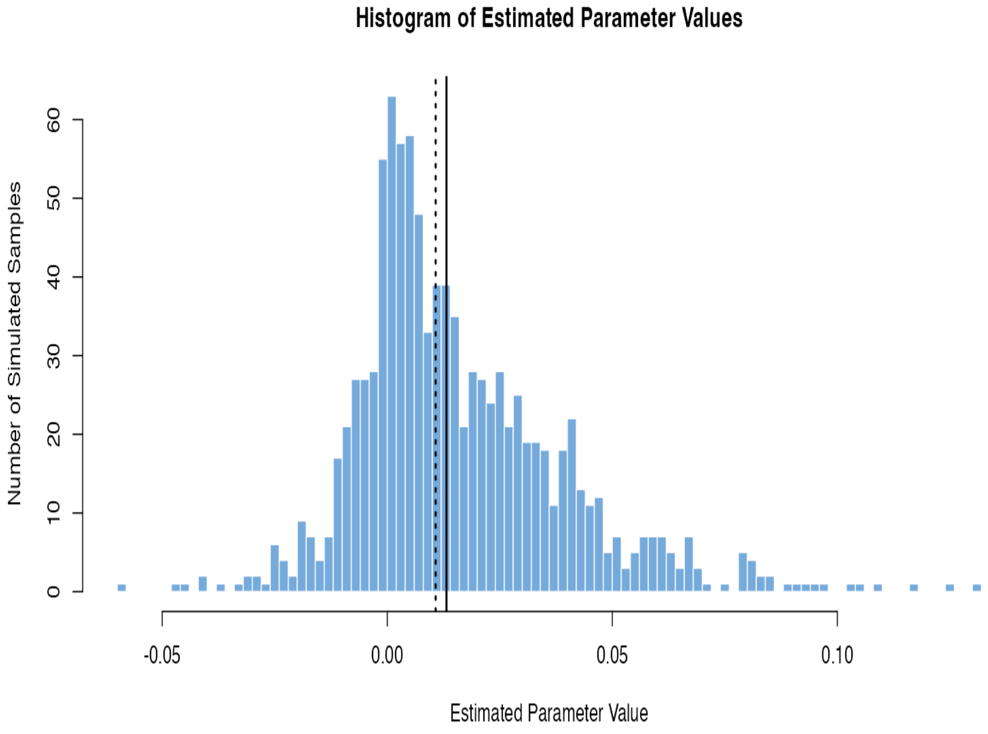

95% of parameter estimates fall within the interval [-0.02, 0.07]. Vertical solid line indicates the population value you set for the parameter; vertical dotted line indicates the median of parameter estimates from the simulated samples.
