# Supplementary material for: From imagination to activism: Cognitive alternatives motivate commitment to activism through identification with social movements and collective efficacy
Source: Br J Soc Psychol. 2024 Nov 15;64(1):e12811. doi: 10.1111/bjso.12811 (PMC11590046; doi:10.1111/bjso.12811)
Supplement: Supplementary file 1 — Data S1. [file BJSO-64-0-s001.zip › Supp 1 measures.docx]

Supplement: Measures

# **Study 1: Climate camp (2019)**

**Response scale for all measures**

*English:*

1 = strongly disagree [1]

2 = [2]
3 = [3]
4 = [4]

5 = [5]
6 = [6]
7 = strongly agree [7]

*German:*

1 = Stimme gar nicht zu [1]

2 = [2]
3 = [3]
4 = [4]

5 = [5]
6 = [6]
7 = Stimme vollkommen zu [7]

**Collective action intention**

*English:*

I am willing to ...

1. ...  take part in demonstrations for climate justice regularly, even if it is very costly for me (e.g. being away many weekends).
2. ...  dedicate myself to a climate justice group on a regular basis, even if it takes up a large part of my free time.
3. ...  participate in collective actions of civil disobedience for climate justice, even if it might have negative consequences for me personally.
4. ...  deliberately break laws, together with others for the sake of climate justice, and live with the criminal consequences.

*German:*

Ich bin bereit ...

1. ...  mich regelmäßig in einer Gruppe für Klimagerechtigkeit zu engagieren, auch wenn es einen großen Teil meiner Freizeit kostet.
2. ...  regelmäßig an Demonstrationen für Klimagerechtigkeit teilzunehmen, auch wenn es für mich sehr aufwändig ist (z.B. viele Wochenenden unterwegs sein).
3. ...  für Klimagerechtigkeit an kollektiven Aktionen zivilen Ungehorsams teilzunehmen, auch wenn es negative Folgen für mich persönlich haben könnte.
4. ...  mich gemeinsam mit anderen für Klimagerechtigkeit bewusst über Gesetze hinwegzusetzen und mit den strafrechtlichen Konsequenzen zu leben.

**Socio-ecological cognitive alternatives**

*English:*

1. I can well imagine what it is like to live in a society based on solidarity.
2. It is clear to me what is necessary to make society socially and ecologically just.
3. I have a clear picture of how a socially and ecologically just society could look like.
4. ~~I don’t know a suitable counter-proposal to the current society. (reversed)~~ (excluded after T2)

*German:*

1. Ich habe ein klares Bild davon, wie eine sozial und ökologisch gerechte Gesellschaft aussehen könnte.
2. Für mich ist deutlich, was es braucht, um die Gesellschaft sozial und ökologisch gerecht zu machen.
3. Ich kann mir gut vorstellen, wie es ist, in einer Gesellschaft zu leben, die auf Solidarität beruht.
4. ~~Ich kenne keinen geeigneten Gegenentwurf zur momentanen Gesellschaft.~~ ~~(reversed)~~ (excluded after T2)

**Collective efficacy**

*English:*

As the climate justice movement, we can ...

1. ...  collectively plan and implement very effective actions for climate justice in the future.
2. ...  collectively change society in a relevant positive way, even if powerful economic interests are against it.
3. ...  collectively make an enormously important contribution to fighting the climate crisis in the next 10 years.
4. ...  collectively make an important contribution to making society more socially and ecologically just in the next 10 years.

*German:*

Als Klimagerechtigkeitsbewegung können wir ...

1. ...  in Zukunft gemeinsam sehr wirkungsvolle Aktionen für Klimagerechtigkeit planen und umsetzen.
2. ...  gemeinsam die Gesellschaft maßgeblich positiv verändern, auch wenn mächtige wirtschaftliche Interessen dagegen sind.
3. ...  zusammen einen enorm wichtigen Beitrag zur Bekämpfung der Klimakrise in den nächsten 10 Jahren leisten.
4. ...  zusammen einen wichtigen Beitrag dazu leisten, die Gesellschaft in den nächsten 10 Jahren sozial und ökologisch deutlich gerechter zu machen.

**Movement identification**

*English:*

1. Ich identifiziere mich stark mit der Klimagerechtigkeitsbewegung.
2. Ich fühle mich mit der Klimagerechtigkeitsbewegung stark verbunden.
3. Ich bin sehr froh, zur Klimagerechtigkeitsbewegung zu gehören.

*German:*

1. Ich identifiziere mich stark mit der Klimagerechtigkeitsbewegung.
2. Ich fühle mich mit der Klimagerechtigkeitsbewegung stark verbunden.
3. Ich bin sehr froh, zur Klimagerechtigkeitsbewegung zu gehören.

# Study 2: Conference ‘Future For All’ (2020)

**Response scale for all measures**

*English:*

1 = absolutely disagree [1]

2 = [2]
3 = [3]
4 = [4]

5 = [5]
6 = [6]
7 = absolutely agree [7]

*German:*

1 = stimmt absolut nicht [1]

2 = [2]
3 = [3]
4 = [4]

5 = [5]
6 = [6]
7 = stimmt absolut [7]

**Collective action intention**

*English:*

For social and/or ecological justice, I am willing ...

1. … to participate in actions of civil disobedience, even if this has negative consequences for me personally.
2. … to do poorly or completely unpaid work for a political organisation, even if this makes my life considerably more strenuous and stressful.
3. … to take part in demonstrations on a regular basis, even if this leaves little free time for me.

*German:*

Für soziale und/oder ökologische Gerechtigkeit bin ich bereit, …

1. … an Aktionen zivilen Ungehorsams teilzunehmen, auch wenn es negative Folgen für mich persönlich hat.
2. … schlecht oder gänzlich unbezahlte Arbeit für eine politische Organisation zu verrichten, auch wenn dies mein Leben deutlich anstrengender und stressiger macht.
3. … regelmäßig an Demonstrationen teilzunehmen, auch wenn dadurch wenig freie Zeit für mich übrig bleibt.

**Socio-ecological cognitive alternatives**

*English:*

1. I have a concrete vision of how a socially and ecologically just society could look like.
2. It is very clear for me how life in a society based on global and local solidarity would be like.
3. I find it difficult to clearly imagine a socially and ecologically just society.

*German:*

1. Ich habe eine konkrete Vision davon, wie eine sozial und ökologisch gerechte Gesellschaft aussehen könnte.
2. Für mich ist sehr klar, wie es wäre, in einer Gesellschaft zu leben, die auf lokaler und globaler Solidarität beruht.
3. Ich finde es schwer, mir eine sozial und ökologisch gerechte Gesellschaft genau vorzustellen.

**Collective efficacy**

*English:*

I am strongly convinced …

1. … that together as social-ecological movements we can decisively push forward the fundamental transformation of our society.
2. … that together as social-ecological movements we can contribute crucially to make the whole world significantly more just.
3. … that through our work together as social-ecological movements we can realize a society based on global and local solidarity.

*German:*

Ich bin fest davon überzeugt, …

1. … dass wir zusammen als sozial-ökologische Bewegungen die fundamentale Transformation unserer Gesellschaft ausschlaggebend vorantreiben können.
2. … dass wir gemeinsam als sozial-ökologische Bewegungen entscheidend dazu beitragen können, die ganze Welt maßgeblich gerechter zu machen.
3. … dass wir durch unsere gemeinsame Arbeit als sozial-ökologische Bewegungen eine Gesellschaft verwirklichen können, die auf globaler und lokaler Solidarität beruht.

**Participative efficacy**

*English:*

I am strongly convinced …

1. … that I, as an individual, can contribute substantially so that we as social-ecological movements can push forward a fundamental societal transformation.
2. … that I, as an individual, can provide an important contribution so that we as social-ecological movements can make the whole world significantly more just.
3. … that I, as an individual, can have a meaningful impact so that we as social-ecological movements can realize a society based on global and local solidarity.

*German:*

Ich bin fest davon überzeugt, …

1. … dass ich als Individuum bedeutend dazu beitragen kann, dass wir als sozial-ökologische Bewegungen eine fundamentale gesellschaftliche Transformation vorantreiben können.
2. … dass ich als Individuum einen wichtigen Beitrag dazu leisten kann, dass wir als sozial-ökologische Bewegungen die ganze Welt maßgeblich gerechter machen können.
3. … dass ich als Individuum einen bedeutsamen Einfluss darauf haben kann, dass wir als sozial-ökologische Bewegungen eine Gesellschaft verwirklichen können, die auf globaler und lokaler Solidarität beruht.

**Movement Identifaction**

*English:*

1. I feel a very strong bond with movements for social-ecological justice.
2. It‘s very fullfilling for me to be part of movements for social-ecological justice.
3. Belonging to movements for social-ecological justice is one of the most important parts of my identity.

*German:*

1. Ich habe eine sehr starke Bindung zu Bewegungen für sozial-ökologische Gerechtigkeit.
2. Es ist für mich sehr erfüllend, zu Bewegungen für sozial-ökologische Gerechtigkeit zu gehören.
3. Die Zugehörigkeit zu Bewegungen für sozial- ökologische Gerechtigkeit, ist einer der wichtigsten Teile meiner Identität.
