# Supplementary material for: From imagination to activism: Cognitive alternatives motivate commitment to activism through identification with social movements and collective efficacy
Source: Br J Soc Psychol. 2024 Nov 15;64(1):e12811. doi: 10.1111/bjso.12811 (PMC11590046; doi:10.1111/bjso.12811)
Supplement: Supplementary file 1 — Data S1. [file BJSO-64-0-s001.zip › Supp 3 detailed regression results.docx]

**Study 1: Climate Camp (2019 )**

- 1. Efficacy path (simple mediation)

|  | | T2-T1 | | | | | ## | T3-T1 | | | | |
| --- | --- | --- | --- | --- | --- | --- | --- | --- | --- | --- | --- | --- |
| Dependent | Predictor | *b* | *SE* | *p* | *b** | 95% CI |  | *b* | *SE* | *p* | *b** | 95% CI |
| Δ Intention | Δ Coll. efficacy | 0.50 | 0.22 | .021* | 0.38 | [0.08, 0.92] |  | 0.49 | 0.23 | .031* | 0.48 | [0.04, 0.94] |
| Δ Coll. efficacy | Δ Alternatives | 0.28 | 0.10 | .008** | 0.41 | [0.07, 0.48] |  | 0.42 | 0.14 | .002** | 0.42 | [0.15, 0.69] |
| Δ Intention | Δ Alternatives | -0.17 | 0.14 | .219 | -0.20 | [-0.45, 0.10] |  | 0.03 | 0.19 | .875 | 0.03 | [-0.34, 0.40] |

- 1. Identification path (simple mediation)

|  | | T2-T1 | | | | | ## | T3-T1 | | | | |
| --- | --- | --- | --- | --- | --- | --- | --- | --- | --- | --- | --- | --- |
| Dependent | Predictor | *b* | *SE* | *p* | *b** | 95% CI |  | *b* | *SE* | *p* | *b** | 95% CI |
| Δ Intention | Δ Identification | 0.57 | 0.15 | < .001*** | 0.69 | [0.29, 0.86] |  | 0.68 | 0.17 | < .001*** | 0.63 | [0.35, 1.01] |
| Δ Identification | Δ Alternatives | 0.47 | 0.14 | .001*** | 0.50 | [0.20, 0.74] |  | 0.42 | 0.16 | .010** | 0.43 | [0.10, 0.73] |
| Δ Intention | Δ Alternatives | -0.34 | 0.18 | .052 | -0.44 | [-0.69, 0.00] |  | -0.11 | 0.16 | .476 | -0.10 | [-0.42, 0.19] |

- 1. Both paths (parallel mediation)

|  | | T2-T1 | | | | | ## | T3-T1 | | | | |
| --- | --- | --- | --- | --- | --- | --- | --- | --- | --- | --- | --- | --- |
| Dependent | Predictor | *b* | *SE* | *p* | *b** | 95% CI |  | *b* | *SE* | *p* | *b** | 95% CI |
| Δ Intention | Δ Coll. efficacy | 0.32 | 0.20 | .122 | 0.26 | [-0.08, 0.72] |  | 0.18 | 0.25 | .468 | 0.17 | [-0.31, 0.68] |
| Δ Coll. efficacy | Δ Alternatives | 0.28 | 0.10 | .008** | 0.41 | [0.07, 0.48] |  | 0.42 | 0.14 | .003** | 0.43 | [0.15, 0.69] |
| Δ Intention | Δ Identification | 0.52 | 0.16 | .001******* | 0.60 | [0.21, 0.83] |  | 0.57 | 0.20 | .004** | 0.53 | [0.18, 0.95] |
| Δ Identification | Δ Alternatives | 0.48 | 0.14 | .001*** | 0.50 | [0.20, 0.75] |  | 0.40 | 0.16 | .010* | 0.42 | [0.09, 0.71] |
| Δ Intention | Δ Alternatives | -0.41 | 0.17 | .019* | -0.50 | [-0.75, -0.07] |  | -0.09 | 0.16 | .568 | -0.09 | [-0.41, 0.23] |

**Study 2: Online-Conference (2020)**

- 1. Efficacy path (simple mediation)

|  | | T2-T1 | | | | | ## | T3-T1 | | | | |
| --- | --- | --- | --- | --- | --- | --- | --- | --- | --- | --- | --- | --- |
| Dependent | Predictor | *b* | *SE* | *p* | *b** | 95% CI |  | *b* | *SE* | *p* | *b** | 95% CI |
| Δ Intention | Δ Coll. efficacy | 0.34 | 0.10 | < .001*** | 0.39 | [0.15, 0.53] |  | 0.24 | 0.09 | .005** | 0.32 | [0.07, 0.41] |
| Δ Coll. efficacy | Δ Alternatives | 0.50 | 0.13 | < .001*** | 0.34 | [0.24, 0.76] |  | 0.11 | 0.14 | .401 | 0.08 | [-0.15, 0.38] |
| Δ Intention | Δ Alternatives | 0.11 | 0.17 | .529 | 0.08 | [-0.23, 0.44] |  | -0.25 | 0.13 | .056 | -0.25 | [-0.51, 0.01] |

- 1. Identification path (simple mediation)

|  | | T2-T1 | | | | | ## | T3-T1 | | | | |
| --- | --- | --- | --- | --- | --- | --- | --- | --- | --- | --- | --- | --- |
| Dependent | Predictor | *b* | *SE* | *p* | *b** | 95% CI |  | *b* | *SE* | *p* | *b** | 95% CI |
| Δ Intention | Δ Identification | 0.54 | 0.14 | < .001*** | 0.59 | [0.27, 0.80] |  | 0.44 | 0.12 | < .001*** | 0.55 | [0.20, 0.68] |
| Δ Identification | Δ Alternatives | 0.37 | 0.14 | .008** | 0.30 | [0.10, 0.65] |  | 0.10 | 0.12 | .415 | 0.09 | [-0.14, 0.35] |
| Δ Intention | Δ Alternatives | 0.06 | 0.17 | .735 | 0.05 | [-0.28, 0.39] |  | -0.24 | 0.12 | .038* | -0.27 | [-0.47, -0.01] |

- 1. Both paths (parallel mediation)

|  | | T2-T1 | | | | | ## | T3-T1 | | | | |
| --- | --- | --- | --- | --- | --- | --- | --- | --- | --- | --- | --- | --- |
| Dependent | Predictor | *b* | *SE* | *p* | *b** | 95% CI |  | *b* | *SE* | *p* | *b** | 95% CI |
| Δ Intention | Δ Coll. efficacy | 0.16 | 0.10 | .126 | 0.19 | [-0.04, 0.36] |  | 0.12 | 0.09 | .193 | 0.18 | [-0.06, 0.29] |
| Δ Coll. efficacy | Δ Alternatives | 0.49 | 0.13 | < .001*** | 0.34 | [0.23, 0.74] |  | 0.11 | 0.14 | .408 | 0.08 | [-0.15, 0.38] |
| Δ Intention | Δ Identification | 0.48 | 0.15 | .002** | 0.52 | [0.18, 0.77] |  | 0.40 | 0.12 | .001** | 0.50 | [0.16, 0.64] |
| Δ Identification | Δ Alternatives | 0.37 | 0.14 | .007** | 0.29 | [0.10, 0.64] |  | 0.09 | 0.12 | .463 | 0.08 | [-0.15, 0.34] |
| Δ Intention | Δ Alternatives | 0.01 | 0.17 | .974 | 0.00 | [-0.32, 0.34] |  | -0.25 | 0.12 | .032* | -0.27 | [-0.48, -0.02] |

- 1. Additional analysis of efficacy related mediators: collective and participative efficacy (parallel mediation)

|  | | T2-T1 | | | | | ## | T3-T1 | | | | |
| --- | --- | --- | --- | --- | --- | --- | --- | --- | --- | --- | --- | --- |
| Dependent | Predictor | *b* | *SE* | *p* | *b** | 95% CI |  | *b* | *SE* | *p* | *b** | 95% CI |
| Δ Intention | Δ Coll. efficacy | 0.35 | 0.11 | .002** | 0.41 | [0.13, 0.57] |  | 0.20 | 0.10 | .057 | 0.27 | [-0.01, 0.40] |
| Δ Coll. efficacy | Δ Alternatives | 0.48 | 0.13 | < .001*** | 0.34 | [0.23, 0.74] |  | 0.14 | 0.13 | .306 | 0.10 | [-0.12, 0.40] |
| Δ Intention | Δ Part. efficacy | 0.00 | 0.09 | .989 | 0.00 | [-0.18, 0.18] |  | 0.09 | 0.10 | .374 | 0.13 | [-0.11, 0.29] |
| Δ Part. efficacy | Δ Alternatives | 0.21 | 0.14 | .133 | 0.14 | [-0.06, 0.49] |  | 0.26 | 0.15 | .079 | 0.17 | [-0.03, 0.54] |
| Δ Intention | Δ Alternatives | 0.11 | 0.16 | .487 | 0.09 | [-0.21, 0.44] |  | -0.27 | 0.14 | .043* | -0.27 | [-0.54, -0.01] |

**Pooled analysis**

|  | | T2-T1 | | | | | ## |
| --- | --- | --- | --- | --- | --- | --- | --- |
| Dependent | Predictor | *b* | *SE* | *p* | *b** | 95% CI |  |
| Δ Intention | Δ Coll. efficacy | 0.24 | 0.05 | < .001*** | 0.28 | [0.15, 0.34] |  |
| Δ Coll. efficacy | Δ Alternatives | 0.26 | 0.05 | < .001*** | 0.24 | [0.16, 0.36] |  |
| Δ Intention | Δ Identification | 0.24 | 0.07 | < .001*** | 0.25 | [0.11, 0.38] |  |
| Δ Identification | Δ Alternatives | 0.22 | 0.05 | < .001*** | 0.23 | [0.13, 0.31] |  |
| Δ Intention | Δ Alternatives | -0.04 | 0.04 | .396 | -0.04 | [-0.13, 0.05] |  |
